# Relationship of body mass index and waist circumference with clinical outcomes following percutaneous coronary intervention

**DOI:** 10.1371/journal.pone.0208817

**Published:** 2018-12-13

**Authors:** Yonggu Lee, Uram Jin, Won Moo Lee, Hong-Seok Lim, Young-Hyo Lim

**Affiliations:** 1 Department of Cardiology, Hanyang University Guri Hospital, Guri, Gyeonggi-do, Republic of Korea; 2 Department of Cardiology, Ajou University School of Medicine, Suwon, Gyeonggi-do, Republic of Korea; 3 Department of Obstetrics and Gynecology, Hanyang University College of Medicine, Seoul, Republic of Korea; 4 Division of Cardiology, Department of Internal Medicine, Hanyang University College of Medicine, Seoul, Republic of Korea; Liverpool John Moores University, UNITED KINGDOM

## Abstract

**Background:**

A biphasic, U-shape relationship has been reported between body mass index (BMI) and clinical outcomes following percutaneous coronary intervention (PCI). However, the relationship between waist circumference (WC) and the cardiovascular risk following PCI has not been reported.

**Methods:**

A prospective cohort study was performed. A major adverse cardiac event (MACE) was defined as a composite of cardiac death (CD), nonfatal myocardial infarction (NFMI) and target vessel revascularization (TVR). Patients were evenly divided into 4 groups according to BMI (Q1_BMI_, Q2_BMI_, Q3_BMI_ and Q4_BMI_) and WC (Q1_WC_, Q2_WC_, Q3_WC_ and Q4_WC_).

**Results:**

A total of 1,421 patients were observed for 5 years. The risk of the composite events of CD and NFMI (CD/NFMI) was lower in the Q3_WC_ and Q4_WC_ groups than in the Q1_WC_ group, whereas it was only marginally lower in the Q2BMI group than in the Q1BMI group (ANOVA, p = 0.062). The risk of MACE was highest in the Q1_WC_ group and lowest in the Q3_WC_ group; however, the risk of MACE did not differ among the 4 groups, according to BMI. Multivariate Cox-regression analyses showed that the risk of CD/NFMI gradually decreased with BMI (linear *p* = 0.030) and with WC (linear *p* = 0.015). The risks of TVR and MACEs that were driven by TVRs showed a distinguishing biphasic, U-shaped relationship with WC (nonlinear *p* = 0.009) but not with BMI (nonlinear *p* = 0.439). Landmark survival analysis showed that the incidences of CD and NFMI were higher in the lower BMI groups and lower WC groups than in the higher BMI groups and higher WC groups, respectively, until 1 year and did not differ afterward. In contrast, the incidence of MACE was highest in Q1_WC_ and lowest in Q3_WC_ (log-rank *p* = 0.003), whereas the incidence was not different among the groups according to BMI.

**Conclusions:**

Both BMI and WC were associated with a lower risk of early episodes of CD and NFMI after PCI. In the late period after PCI, WC demonstrated a biphasic, U-shaped association between cardiovascular outcomes and adiposity, whereas BMI did not.

## Introduction

Obesity is a well-known risk factor for cardiovascular disease (CVD) and mortality in the general population [1–3]. Obesity is associated with worse major cardiovascular risk factors, including lipid profiles, blood pressure and glucose levels; it also accelerates atherosclerotic processes and increases cardiovascular burden, which may lead to systolic and diastolic ventricular dysfunction [4, 5]. However, the paradoxical beneficial effects of mild to moderate obesity on clinical outcomes have been repeatedly reported in cardiovascular research [6]. Large-scale, population-based studies have shown J-curve relationships between body mass index (BMI) and the risk of CVDs and death [7]. Several studies have also reported a negative association between BMI and the rate of cardiovascular events after percutaneous coronary intervention (PCI) [8, 9]. One criticism of the paradoxical beneficial effect demonstrated in those studies is that high BMI may reflect not only high adiposity but also an abundance of skeletal muscles, which has been reported to be associated with lower mortality rates [10–13]. Waist circumference (WC), which indicates central adiposity and reflects little lean body mass, has been reported to be a better predictor of cardiovascular events and mortality than is BMI [14]. However, no study has reported a comparison of WC and BMI in relation to the clinical outcomes following PCI. Therefore, we comparatively investigated the influences of BMI and WC on cardiovascular outcomes in a prospective PCI registry.

## Methods

### Patients

From January 2009 to December 2013, a prospective cohort study was performed at two high-volume PCI centers located in Seoul and Suwon, Republic of Korea. Patients undergoing PCI with drug-eluting stents (DESs) were consecutively enrolled in the registry. Written informed consent was obtained from all patients before their enrollment in the study. The study protocol complied with the principles expressed in the Declaration of Helsinki. The Institutional Review Board of Hanyang University Medical Center approved the study protocol and monitored the study process (IRB No. HYUH 2017-11-031). Patients younger than 35 years of age, patients who had previously undergone coronary artery bypass surgery, patients who suffered from debilitating conditions, including advanced malignancies, advanced liver cirrhosis, end-stage renal disease, severe autoimmune diseases and cerebrovascular accidents with major sequelae, and patients with a life expectancy of less than 12 months were excluded. Information regarding demographic characteristics, past medical histories and social histories was obtained from all patients before they were registered. Lipid profiles, serum glucose levels and hemoglobin A1c levels were measured the morning before the index PCI, after the patients had fasted for 8 hours. The estimated glomerular filtration rate (eGFR) was calculated using the Modification of Diet in Renal Diseases study equation, and chronic kidney disease (CKD) was defined as an eGFR ≤60 ml/min/1.73 m^2^. Before the procedure, 300 mg of aspirin and P2Y12 inhibitors (600 mg of clopidogrel, 60 mg of prasugrel or 180 mg of ticagrelor) were administered to all patients. After the index PCI, all patients received dual antiplatelet agent therapy (DAPT) for ≥1 year and high-intensity statin therapy indefinitely, unless they had contraindications to statin use.

### Measurements of height, weight and waist circumference

Body weight, height and WC were usually determined on the day before the index PCI. In patients with acute coronary syndrome (ACS) and who had presented in emergency settings, body size measurements were obtained within 7 days after the index PCI. WC was measured in the mid portion between the lowest margin of the ribs and the highest part of the iliac crest when the patients were in a supine position and after the expiration of a breath.

### PCI and the definitions of clinical outcomes

PCI was performed using standard techniques. A stenotic lesion that required PCI was defined as a lesion with luminal narrowing ≥70%, based on quantitative angiographic measurements. During the observation period, repeat coronary angiography was performed when patients presented with symptoms of angina or signs of myocardial ischemia. Standardized definitions of clinical events frequently used in cardiovascular trials were used, as described by Hicks et al [15]. Cardiac death (CD) included death resulting from acute myocardial infarction (MI), heart failure or cardiogenic shock. Myocardial infarction (MI) was defined as an increase in cardiac troponin I levels to a level at least 3 times the upper normal limit, with one or more clinical indicator of myocardial ischemia, including symptoms of angina, ST-segment changes on electrocardiography and wall motion abnormalities on echocardiography. Nonfatal MI (NFMI) was defined as an MI that did not result in CD. Target vessel revascularization (TVR) was defined as the performance of balloon angioplasty or stent implantation in the target vessel during the observation period. TVR was performed when a lesion with luminal narrowing ≥70% or functional ischemia was present in the target vessel territory on any diagnostic test. A major adverse cardiac event (MACE) was defined as a combination of CD, NFMI and TVR. PCIs for *de novo* stenotic lesions in nontarget coronary arteries were not considered to be TVR, and the first event that occurred during the observation period was used as the clinical event of a patient.

### Statistical analysis

The patients were equally divided into 4 groups, according to BMI (Q1_BMI_, Q2_BMI_, Q3_BMI_ and Q4_BMI_) and WC (Q1_WC_, Q2_WC_, Q3_WC_ and Q4_WC_). Analysis of variance (ANOVA) was used to compare continuous variables, and a Chi-squared test was used to compare categorical variables among the 4 groups, based on each obesity index. A Kruskal-Wallis test was employed for variables with a skewed distribution. Kaplan-Meier survival analysis with a log-rank test was used to compare cumulative incidences of the clinical events among the 4 groups, based on BMI and WC. A 1-year landmark analysis was performed to evaluate the different influences of obesity indexes on short-term and long-term outcomes. Multiple Cox regression analysis with a stepwise variable selection process was performed to estimate the difference in the risk of clinical events among the 4 groups, based on BMI and WC. The nonlinear relationship between the obesity indexes as continuous variables and the risk of clinical events was evaluated using multiple Cox regression models, with a restrictive cubic spline function for the obesity indexes to evaluate changes in the pattern of associations between the obesity indexes and clinical outcomes. Comparison between a linear model and a nonlinear model was performed using ANOVA. Age, sex, diabetes mellitus, hypertension, CKD, current smoking, prior PCI, the diagnosis of ST-segment elevation MI (STEMI), the duration of DAPT, the number of narrowed coronary arteries, stent type (1st vs. 2nd generation DES), total stent length, average stent diameter and complete revascularization were included in the multiple Cox regression models as covariates. All statistical analyses were performed using R-3.4.0 (from the R foundation for Statistical Computing), and *p* <0.05 was considered to be statistically significant.

## Results

### Baseline characteristics

A total of 1,421 patients were observed for 3,390 person-years (2.38 years/person). The baseline and angiographic characteristics of the entire study population and patients in each BMI and WC group are described in Table 1 and S1 Table. Patient age decreased with BMI but not with WC. The prevalence of hypertension increased with both BMI and WC, whereas the prevalence of diabetes mellitus increased only with WC. The frequency of ACS decreased with BMI; however, it did not differ based on WC. The serum levels of hemoglobin, total cholesterol, LDL cholesterol, triglyceride and hemoglobin A1c increased with both BMI and WC. The left ventricular ejection fraction increased with BMI and WC; however, the increments among the groups were negligible. The number of narrowed coronary arteries, rate of complete revascularization, number of stents implanted, average stent diameter, total stent length, stent types and frequency of 2nd generation DES use did not differ based on BMI and WC. There was a moderate correlation between BMI and WC (*r* = 0.68, *p* <0.001).

**Table 1:** Baseline and angiographic characteristics of the study population.

	N = 1421
Clinical characteristics	
Age (years)	63.5 ± 10.6
Male sex	949 (66.8%)
Center 2	453 (31.9%)
Diabetes mellitus	465 (32.7%)
Hypertension	879 (61.9%)
CKD (eGFR <60 mL/min/1.73 m^2^)	236 (16.6%)
Current smoking	367 (25.9%)
Past history of PCI	233 (16.4%)
Clinical diagnosis	
Stable angina	399 (28.1%)
Unstable angina	744 (52.4%)
NSTEMI	183 (12.9%)
STEMI	95 (6.7%)
Systolic blood pressure (mmHg)	134.3 ± 23.0
Diastolic blood pressure (mmHg)	77.4 ± 12.0
Body mass index (kg/m^2^)	24.9 ± 3.0
Waist circumference (cm)	89.8 ± 8.1
Waist-Height ratio	0.55± 0.06
Laboratory tests	
Hemoglobin (g/dl)	13.4 ± 1.8
Total cholesterol (mg/dl)	168.1 ± 40.0
LDL cholesterol (mg/dl)	96.0 (75.0, 119.0)
HDL cholesterol (mg/dl)	40.0 (34.0, 47.7)
Triglyceride (mg/dl)	117.0 (84.0, 169.0)
HgA1c (%)	6.1 (5.7, 6.9)
hsCRP (mg/dl)	0.12 (0.05, 0.42)
eGFR (ml/min/1.73 m^2^)	77.3 ± 18.3
Post PCI medications	
Statin	1192 (84.1%)
Angiotensin blockers	1050 (74.0%)
Beta-blockers	893 (63.0%)
LVMI (kg/m^2^)	102.8±32.2
LV ejection fraction (%)	68.8 ± 9.2
Duration of DAPT (months)	18 (11, 34)
Duration of follow-up (months)	29 (13, 41)
Angiographic characteristics	
Number of coronary arteries narrowed	
1	818 (57.8%)
2	428 (30.2%)
3	170 (12.0%)
Coronary arteries involved	
LMCA	84 (5.9%)
LAD	992 (70.1%)
LCX	505 (35.7%)
RCA	594 (41.9%)
Complete revascularization	1258 (88.6%)
Number of stents implanted	
1	645 (45.6%)
2	370 (26.1%)
≥3	401 (28.3%)
Average stent diameters (mm)	3.1 (3.0, 3.5)
Total stent length (mm)	41 (24, 71)
Types of stents	
Paclitaxel	245 (17.3%)
Sirolimus	321 (22.7%)
Everolimus	878 (62.0%)
Others	17 (1.2%)
Second generation DES	892 (63.0%)

Data are shown as the mean ± SD or n (%).

Data with a skewed distribution are presented with the median (interquartile range)

CKD, chronic kidney diseases; PCI, percutaneous coronary intervention; LDL, low density lipoprotein; HDL, high density lipoprotein; hsCRP, highly sensitive C-reactive protein; eGFR, estimated glomerular filtration rate; LMCA, left main coronary artery; LAD; left anterior descending artery; LCX, left circumflex artery; RCA right coronary artery; DES, drug eluting stent.

### Comparison of clinical outcomes among the groups

**Fig 1:**
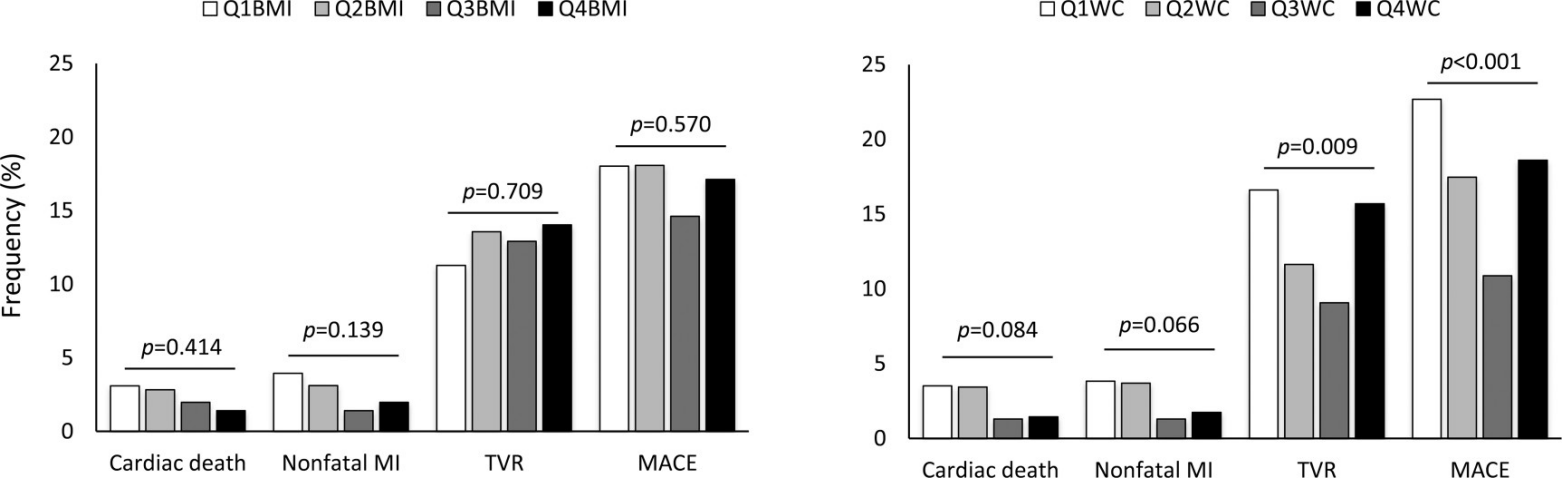
Overall incidence of cardiac events after PCI, based on obesity indexes. There were no differences in the overall frequencies of CD, NFMI, TVR and MACE among the groups, based on BMI. The occurrences of CD and NFMI were marginally more frequent in the Q1_WC_ and Q2_WC_ groups than were those in the Q3_WC_ and Q4_WC_ groups, whereas the overall incidences of TVRs and MACEs were lowest in the Q3_WC_ group and showed biphasic relations with WC.

The median observation duration was 29 months (interquartile range: 13–41 months). The overall incidences of CD, NFMI, TVR and MACEs did not differ based on BMI. The overall incidences of TVR and MACEs were lowest in the Q3_WC_ group (among the WC groups), whereas the overall incidences of CD and NFMI did not differ, based on WC (Fig 1 and S2 Table).

**Fig 2:**
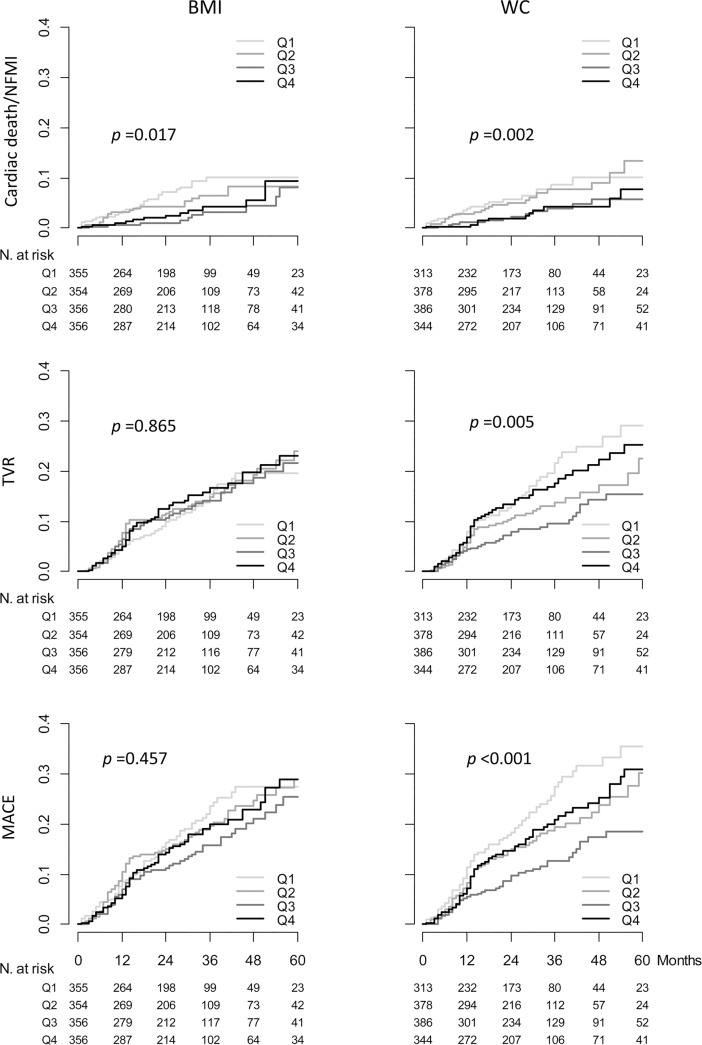
Cumulative incidence of cardiac events after PCI based on obesity indexes. CD/NFMI occurred more frequently in the high WC and high BMI groups than in the low WC and low BMI groups. In contrast, there were no differences in the risk of TVR and MACEs among the groups, based on BMI. However, the incidence of TVR and MACEs was significantly different among the groups (based on WC), and the incidence was highest in the Q1_WC_ group and lowest in the Q3_WC_ group. CD, cardiac death; NFMI, nonfatal myocardial infarction; CD/NFMI, composite of CD and NFMI; TVR, target vessel revascularization; MACE, major adverse cardiac event; BMI body mass index; WC, waist circumference.

Kaplan-Meier survival curve analysis showed that the occurrence of CD and NFMI did not differ among the groups, based on BMI. CD was more frequent in the Q1_WC_ and Q2_WC_ groups than in the Q3_WC_ and Q4_WC_ groups. The occurrence of NFMI was marginally more frequent in the Q1_WC_ and Q2_WC_ groups than in the Q3_WC_ and Q4_WC_ groups (S1 Fig). The composite events of CD and NFMI (CD/NFMI) were most frequent in the Q1_BMI_ and Q1_WC_ groups and least frequent in the Q3_BMI_ and Q3_WC_ groups. The occurrence of TVR and MACEs did not differ among the BMI groups; however, it was highest in the Q1_WC_ group and lowest in the Q3_WC_ group among the groups stratified by WC (Fig 2).

**Fig 3:**
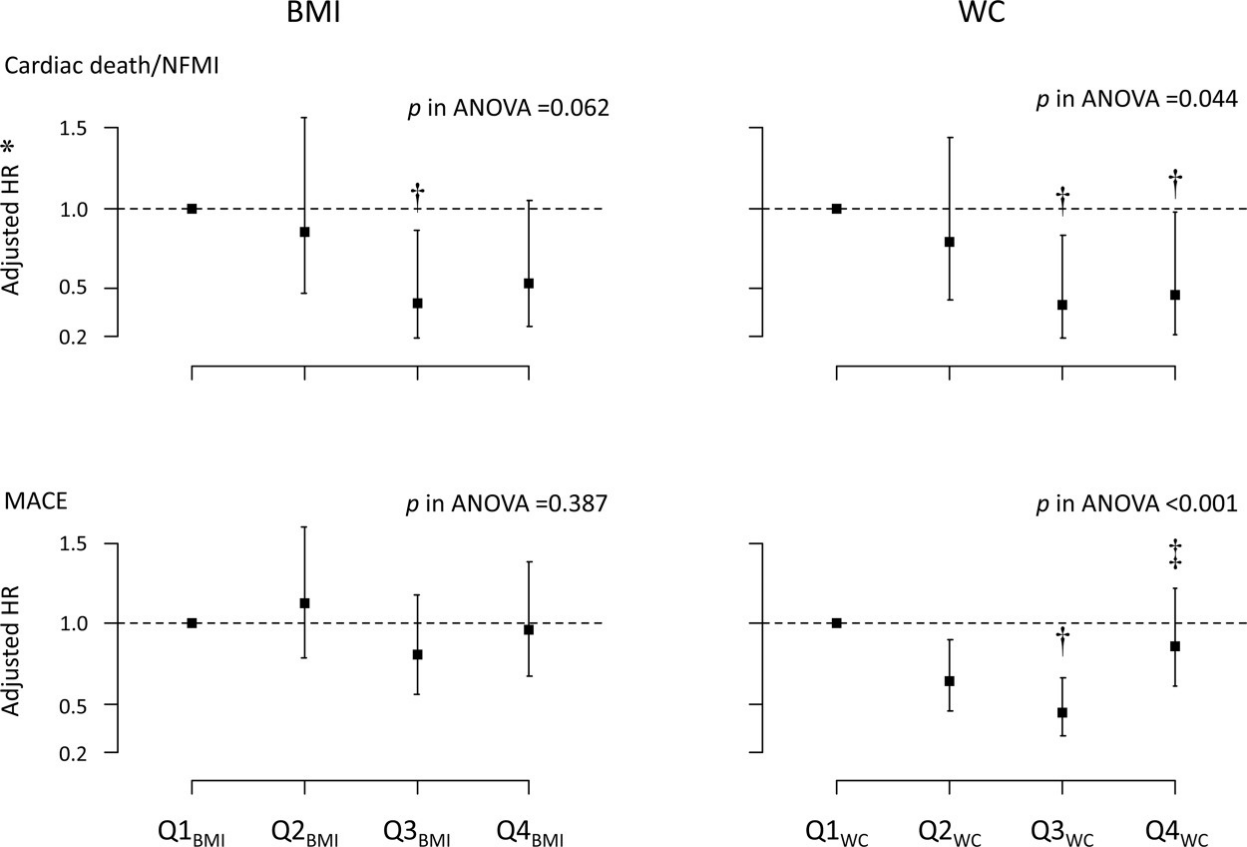
The risks of CD/NFMI and MACEs stratified by the BMI and WC groups. The risk of CD/NFMI decreased in the groups with high obesity indexes; however, the difference was not significant among the BMI groups. The risk of MACEs was not different among the groups stratified by BMI; however, it sequentially decreased in the Q2_WC_ and Q3_WC_ groups and then increased in the Q4_WC_ group. * The hazard ratios were derived from a multiple Cox regression analysis with covariates that included age, sex, diabetes mellitus, hypertension, current smoking, chronic kidney disease, history of PCI, STEMI, stent type (1st vs. 2nd generation), center, total stent length, average stent diameter and complete revascularization and the duration of DAPT. Each model was reduced through a backward variable selection process. † *p* <0.05 vs. Q1 and ‡ *p* <0.05 vs. Q3.

Multiple Cox regression analysis showed that the risk of CD/NFMI was lower in the Q3_BMI_ group than in the Q1_BMI_ group, but there was no significant trend for the risk of CD/NFMI among the 4 BMI groups. The risk of CD/NFMI was lower in the Q3_WC_ and Q4_WC_ groups than in the Q1_WC_ groups. The risk of MACEs was not significantly different among the BMI groups, whereas the risk of MACEs was lower in the Q3_WC_ groups than in the Q1_WC_ and Q4_WC_ group (Fig 3).

**Fig 4:**
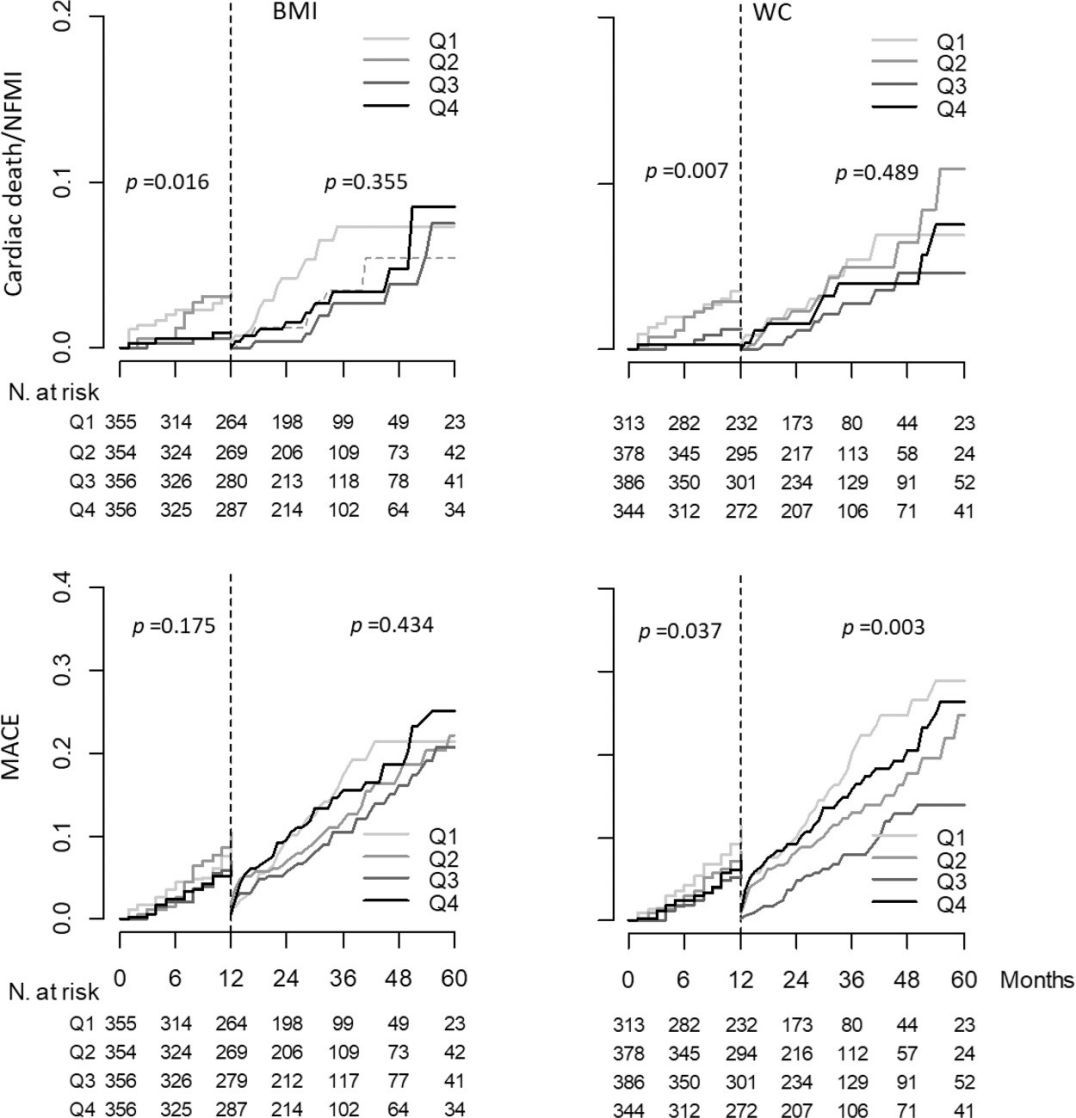
Landmark analysis for the risks of CD/NFMI and MACEs. The risk of CD and NFMI was higher in the groups with low obesity indexes (Q1 and Q2 of both BMI and WC) until 1 year of observation; however, it was not different among the groups stratified by either BMI or WC after 1 year of observation. BMI did not influence the risk of MACEs throughout the observation period. In contrast, the risk of MACEs was significantly higher in the Q1_WC_ group than in the Q3_WC_ group throughout the observation period (11.5% vs. 5.3%, *p* = 0.005). The risk of MACEs was not different between the Q3_WC_ and Q4_WC_ groups until 1 year of observation (5.3% vs. 7.1%, *p* = 0.332); however, it was higher in the Q4_WC_ group after 1 year of observation (13.4% vs. 25.5%, *p* = 0.004). CD, cardiac death; NFMI, nonfatal myocardial infarction; MACE, major adverse cardiac event; BMI body mass index; and WC, waist circumference.

Kaplan-Meier curve analysis with a 1-year landmark showed that CD/NFMI more frequently occurred in the low-BMI (Q1_BMI_ and Q2_BMI_) and low-WC (Q1_WC_ and Q2_WC_) groups compared to the high-BMI (Q3_BMI_ and Q4_BMI_) and high-WC (Q3_WC_ and Q4_WC_) groups, whereas there was no difference in the occurrence of CD/NFMI among the groups stratified by BMI and by WC 1 year after the index PCI. In contrast, there was no difference in the occurrence of MACEs in the groups stratified by BMI 1 year before or after the index PCI, whereas MACEs occurred more frequently in the Q1_WC_ group than in any other groups stratified by WC, and MACEs occurred least frequently in the Q3_WC_ group, both 1 year before and after the index PCI (Fig 4).

### Relationship between the obesity indexes and clinical outcomes

**Fig 5:**
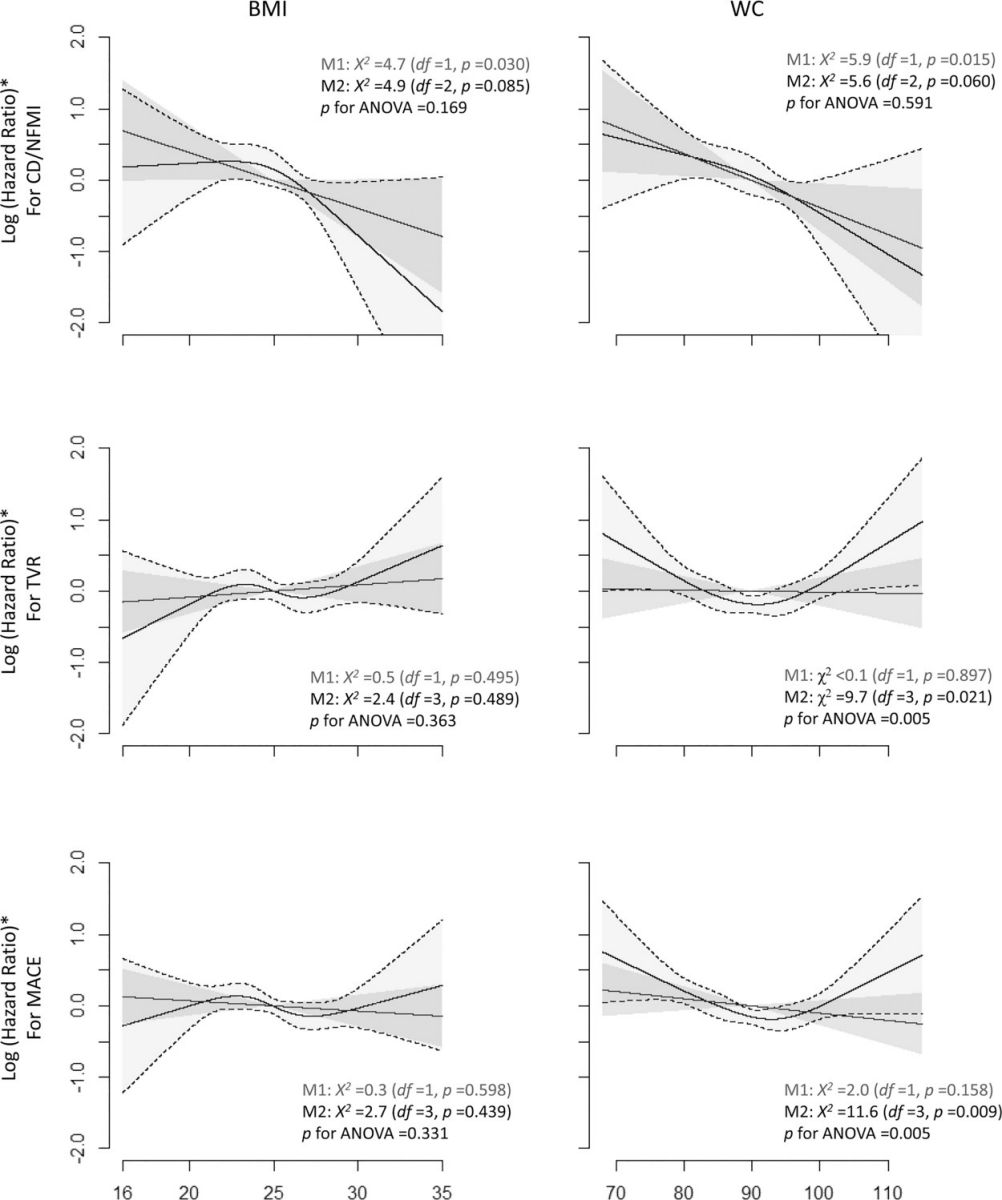
Nonlinear multiple Cox regression models for the risk of clinical events based on BMI and WC. The blue lines indicate linear models (M1), and the black lines indicate nonlinear models with restrictive cubic spline fits (M2). M1 and M2 were compared using ANOVA. The risk of CD and NFMI was linearly associated with the increase in both BMI and WC. The risk of TVR and MACE was not associated with BMI in both the linear and nonlinear model. There were U-shape biphasic associations of WC with the risk of both TVR and MACE, whereas TVR and MACE were not linearly associated with WC. * Adjusted for age, sex, diabetes mellitus, hypertension, current smoking, chronic kidney disease, history of PCI, STEMI, stent type (1st vs. 2nd generation), center, TSL, average stent diameter, complete revascularization and the duration of DAPT. Each model was reduced by a backward variable selection process. CD, cardiac death; NFMI, nonfatal myocardial infarction; MACE, major adverse cardiac event; BMI body mass index; and WC, waist circumference; *df*, degrees of freedom.

The multiple Cox regression models with a nonlinear restricted cubic spline fit for the obesity indexes showed that simulated log-transformed hazard ratios (LogHRs) for CD/NFMI were only linearly associated with both BMI and WC. The simulated LogHRs for TVR and MACEs were not significantly associated with BMI in the model. In contrast, the LogHRs for TVR and MACEs gradually decreased until WC values indicating mild central obesity were reached (WC of 91 cm for TVR and 93 cm for MACE), and then the values gradually increased thereafter, and the U-shape biphasic regression spline fit for WC significantly improved the performance of the whole models predicting for both the risk of TVR and that of MACE (Fig 5).

Multiple Cox regression analyses showed that the risk of CD/NFMI was significantly associated with WC and marginally associated with BMI, with a linear assumption but not with a nonlinear assumption. In contrast, the risk of MACE was significantly associated with WC in both linear and nonlinear assumptions; however, it was not significantly associated with BMI (Table 2).

**Table 2:** Multivariate Cox-regression analysis with restrictive cubic spline fits for obesity indexes.

Model summary		Variables	coefficient	S.E.	χ^2^	d.f.	*p*
CD/NFMI	Model 1	BMI linear	-0.088	0.044	4.0	1	0.0468
	C-statistic	nonlinear	-	-	1.4	1	0.2471
	0.855	Diabetes mellitus	0.730	0.253	8.4	1	0.0039
		Complete revascularization	-0.920	0.296	9.7	1	0.0019
		CKD (eGFR ≤60 mL/min/1.72 m^2^)	1.132	0.254	19.8	1	<0.0001
		Duration of DAPT (months)	-0.080	0.012	44.9	1	<0.0001
	Model 2	WC linear	-0.038	0.017	4.9	1	0.0273
	C-statistic	nonlinear	-	-	0.3	1	0.5800
	0.850	Diabetes mellitus	0.754	0.253	8.9	1	0.0029
		Complete revascularization	-0.940	0.296	10.1	1	0.0015
		CKD (eGFR ≤60 mL/min/1.72 m^2^)	1.161	0.255	20.7	1	<0.0001
		Duration of DAPT (months)	-0.079	0.012	49.3	1	<0.0001
MACE	Model 1	BMI linear	-0.005	0.022	0.1	1	0.8189
	C-statistic	nonlinear	-	-	2.2	2	0.5571
	0.843	Diabetes mellitus	0.348	0.137	6.5	1	0.0109
		Total stent length (per 10 mm)	0.065	0.017	15.1	1	0.0001
		Center 1	-1.102	0.209	27.7	1	<0.0001
		Second generation DES	-1.220	0.195	39.0	1	<0.0001
		Duration of DAPT (months)	-0.072	0.006	142.7	1	<0.0001
	Model 2	WC linear	-0.010	0.008	1.7	1	0.1936
	C-statistic	nonlinear	-	-	9.6	2	0.0085
	0.845	Sex	0.415	0.149	7.8	1	0.0053
		Diabetes mellitus	0.433	0.136	10.2	1	0.0014
		Total stent length (per 10 mm)	0.065	0.017	15.3	1	0.0001
		Center 1	-1.030	0.208	24.6	1	<0.0001
		Second generation DES	-1.120	0.195	37.7	1	<0.0001
		Duration of DAPT (months)	-0.073	0.006	141.4	1	<0.0001

The models were reduced by a backward variable selection process (cut-off point, p <0.05).

CD, cardiac death; NFMI, nonfatal myocardial infarction; MACE, major adverse cardiac event; BMI, body mass index; WC, waist circumference; CKD, chronic kidney disease; eGFR, estimated glomerular filtration rate.

When BMI and WC were included together as linear fits in the multivariate Cox-regression models, the associations of both BMI and WC with the risk of CD/NFMI were weakened by one another’s presence, although WC showed a slightly stronger association (χ^2^ = 1.73, *p* = 0.188) than did BMI (χ^2^ = 0.55, *p* = 0.459; S3 Table). However, the presence of BMI in the model did not weaken the association between WC and the risk of MACE, whereas BMI was not associated with the risk of MACE (S2 Fig. and S3 Table). There was no significant interaction between BMI and WC, in terms of their relationship with the risk of both CD/NFMI (*p* = 0.488) and MACE (*p* = 0.667; S4 Table).

## Discussion

In this study, a beneficial effect of mild to moderate obesity on CD/NFMI was evident using both obesity indexes, BMI and WC, although it was more obvious with WC. In contrast, a beneficial effect on TVR-driven MACEs was not observed when mild obesity was defined by BMI; however, such an effect was observed when it was defined by WC. The lower CD/NFMI rates were observed in obese patients, based on both BMI and WC, until 1 year after the index PCI. However, the beneficial effect of mild central obesity defined by WC was observed on MACE throughout the observation duration. The risk of CD/NFMI was linearly associated with both obesity indexes, BMI and WC. There was a U-shaped relationship between the risk of TVR/MACE and WC, whereas there was no relationship between the risk of TVR/MACEs and BMI.

Although obesity has been an established risk factor for CVDs for a long time, studies have repeatedly shown that obesity was associated with better prognosis in patients with CVDs [6]. Beneficial effects of obesity on clinical outcomes have also been reported in many studies of patients undergoing PCI [8]. A meta-analysis including studies from the bare-metal stent era showed a U-shaped relationship between BMI and mortality in patients undergoing PCI [16]. In a recent large-scale cohort study from the U.K., a negative association between BMI and mortality after DES implantation was observed [17]. However, debates arose because most studies reporting these beneficial effects of obesity used BMI as the obesity index. In fact, many studies have shown that central obesity or abdominal adiposity, as measured by WC or the waist-to-hip ratio, was linearly associated with a higher risk of mortality after PCI [18]. Several studies have also reported that low BMI and central obesity are associated with a high mortality rate among patients with MI [19–21], implying that BMI is not an adequate indicator of true adiposity.

Our results showed that higher BMI was linearly associated with a lower risk of CD and NFMI but not associated with a risk of TVR and TVR-driven MACEs. Similarly, Park et al.[8] reported that the incidence of all-cause deaths and MACEs, including CD, NFMI, stent thrombosis and stroke, decreased as BMI increased. Several studies have reported no association between TVR and BMI after a short-term observation [22]. Increasing WC was also linearly associated with a lower risk of CD or NFMI. However, unlike BMI, WC had a U-shape biphasic relationship with TVR/MACEs. The increased risk of TVR/MACEs in patients with severe central obesity is consistent with the classical notion of central obesity as a risk factor for CVD and mortality. In contrast, a negative association between the risk of CD/NFMI and WC and a decreased risk of TVR in patients with mild central obesity may be unexpected, given that many studies have reported that central obesity is a predictor of cardiovascular mortality in patients with CAD [18]. The relationship between WC and cardiovascular outcome after PCI has been less frequently reported than that of BMI has, and to date, no study has reported an association between high WC and better cardiovascular outcomes after PCI. However, high adiposity itself has been reported as a predictor of good prognosis among patients with CADs. Lavie et al.[12, 23] reported that a high percentage of body fat, which was measured using the sum of the skinfold method, was associated with a lower mortality rate among patients with stable CAD. They suggested that the obesity paradox may also apply to central obesity, as high body fat content, lean body mass and fitness were the actual contributors to the obesity paradox in patients with CAD [24, 25]. Our results may also serve as an evidence against criticism stating that the obesity paradox is misleading because BMI is not an adequate indicator of true adiposity. A high WC at the time of the index PCI may exert not only beneficial effects on clinical outcomes after PCI (as observed with a high BMI) but also might be a better predictor of TVR than is BMI.

The correlation between BMI and WC in our study was not strong (*R*^2^ = 0.47) compared with that in a previous study (*R*^2^ = 0.61) [26]. This result is likely associated with a difference in the body compositions of the population in which the studies were performed. The mean BMI and WC levels were lower (24.8 kg/m^2^ and 90 cm, respectively) in our cohort than in a previous study (30.7 kg/m^2^ and 108 cm, respectively), which means that not many severely obese patients were included in this study. Thus, BMI may reflect a lean body mass, which is associated with a better prognosis in patients with CAD, which may explain why there was no distinct association between TVR and BMI in our results.

Kaplan-Meier curve analysis with 12-month’s landmark showed that the occurrence of CD and NFMI was significantly different, based on both BMI and WC in the early period of observation but not in the late period. In contrast, TVR and MACEs showed a distinctive biphasic relationship with WC after 1 year of observation, particularly TVR, which demonstrated significantly different event rates only 1 year after the index PCI. A recent meta-analysis also showed that the beneficial effects of morbid obesity in patients with established CAD did not last in long-term follow-up periods [27]. Our results may also imply that there are two different underlying reasons for the beneficial effects of obesity on CD/NFMI in the early period and TVR-driven MACE in the late period of observation.

One explanation for these results may be found in our study population who underwent PCI. The classical notion of WC predicting CVD and mortality originated from large population-based studies, in which the cardiovascular events were less frequent than they were in studies of patients with advanced CAD, such as ours, and most cardiovascular events were related to the natural progression of atherosclerotic plaque. However, unlike a general population, patients undergoing PCI have risk factors for CAD progression, such as hypertension, diabetes and smoking more frequently, and they also have various clinical features that could worsen their clinical outcomes, including elevated inflammation, reduced myocardial function, low exercise capacity and poor medication compliance. Therefore, higher metabolic reserves and less cachexia would likely outweigh the cardiovascular consequences of obesity within 1 year of the index PCI, which is not sufficient for atherosclerosis to progress but is sufficient for left ventricular remodeling and heart failure to occur. Small volumes of distribution may also be associated with higher bleeding risk, thereby increasing the early cardiovascular event rates in nonobese patients, shortly after they were introduced to DAPT. Numasawa et al.[28] reported more cases of in-hospital complications after PCI in patients with low BMI because of the higher risk of bleeding. Although BMI may reflect lean body mass rather than reflecting adiposity, and WC primarily presents as central adiposity, both adiposity and lean body mass could serve as metabolic reserves. These factors may explain the negative association between the risk of CD/NFMI and both obesity indexes. The fact that the associations between BMI and WC and the risk of CD/NFMI were weakened by one another’s presence, without significant interactions with one another in the multivariate Cox-regression model, may also indicate the similarity in the underlying mechanism for the association between low BMI and WC and the risk of CD/NFMI.

The biphasic relationship between TVR/MACEs and WC after 1 year of observation may be explained by the high positive remodeling rate of coronary arteries in obese patients. Unlike cardiovascular events occurring through slow atherosclerotic process in the general population, cardiovascular events occurring in patients who underwent PCI are most frequently associated with in-stent restenosis through rapidly progressing neo-atherosclerosis. Using intravascular ultrasonography, Kang et al.[29] determined that obese patients tended to have larger coronary reference diameters and undergo PCI with larger diameter stents. Kim et al.[30] also reported that insulin resistance was associated with high positive remodeling rates. Given that most TVR results from in-stent restenosis and that TVR rates differed only in the late period of observation, the pro-atherosclerotic effects of obesity and larger luminal gains after PCI may balance out, thereby leading to the observation of the lowest risk of TVR in patients with mild central obesity. Furthermore, the higher rates of optimal medical therapy may also contribute to a lower risk of TVR/MACEs in patients with mild central obesity. Studies have reported that optimal medical therapies are more frequently administered to obese patients than to nonobese patients, due to high blood pressure levels and lipid profile levels [31].

To date, many studies have reported the impact of obesity defined by a single measurement of BMI or WC on clinical outcomes after PCI. However, because the body composition and adiposity of patients may change during the observation periods, and because the changes may be more closely related to differences in outcome, it is difficult to make any practical lifestyle recommendations about body weight to patients after PCI using data from these studies. Although few longitudinal data are currently available, in a small-scale retrospective study, Kang et al.[32] reported that weight loss >5% was associated with worse outcomes in patients with acute MI after successful PCI. Larger-scale prospective studies are necessary to develop a practical recommendation regarding obesity for patients undergoing PCI.

### Limitations

This study has several limitations. First, only two high-volume PCI centers located around Seoul participated in this study; therefore, a referral bias may be present. The prevalence of obesity (and other cardiovascular risk factors) and the event rates may not represent those of the entire population undergoing PCI in South Korea. Second, only a small number of patients with a high BMI or WC were included because of the low incidence of obesity in a typical Asian population compared with a typical Western population. The total sample size was also relatively small in our study, compared with those in population-based studies and other multicenter registry studies. Third, the obesity indexes were measured only once; therefore, important changes in the levels of obesity may have occurred during the long observation period and may have caused the differences in outcomes among the groups. Fourth, we did not measure body composition and hip circumference, which may help fortify or disprove our findings. Finally, we did not include any measurements that may provide some mechanistic insights on the paradoxical beneficial effects of obesity, other than the landmark analysis. Therefore, we cannot make any recommendations regarding body weight or WC for patients undergoing PCI based on the findings from this study.

### Conclusion

In patients undergoing PCI, both obesity indexes, BMI and WC, were associated with a lower risk of CD/NFMI, although WC may be a slightly better indicator of the beneficial effects of obesity than is BMI. The risk of CD/NFMI was linearly associated with both BMI and WC; however, the biphasic U-shaped relationship was observed only between the risk of TVR-driven MACE and WC. Our results suggest that the better cardiovascular outcomes observed in obese patients undergoing PCI were not associated with high BMI, reflecting high lean body mass, but were associated with true adiposity and that WC may be a better indicator of the paradoxical beneficial effects of obesity than is BMI in patients undergoing PCI. The differences in the relationships of both BMI and WC with the clinical outcomes, according to time, may also suggest that the reasons underlying the beneficial effects of obesity may differ between the early and the late period of observation. Studies including serial measurements of the obesity indexes are required to determine the underlying causes of the beneficial effect of obesity and produce a recommendation for lifestyle modifications for obese patients undergoing PCI.

## Supporting information

S1 Fig Cumulative incidences of CD and NFMI after PCI based on obesity indexes. CD occurred more frequently in the high WC groups (Q3_WC_ and Q4_WC_) than in the low WC groups (Q1_WC_ and Q2_WC_), whereas there was no difference in the risk of CD among the groups, according to BMI. The incidence of NFMI was only marginally higher in the low WC and BMI groups.
CD, cardiac death; NFMI, nonfatal myocardial infarction; BMI body mass index; WC, waist circumference.
(TIF)

S2 Fig Three-dimensional depiction of the relationship of the risk of clinical events with both BMI and WC. The opaquer sheaths represent the surfaces of log (HR) from multivariate Cox-regression, and the less opaque sheaths represent the surfaces of upper and lower 95% confidence interval limits. The upper panel (green sheaths) represents the risk of MACE, and the lower panel (purple) represents the risk of CD/NFMI. HRs were derived from multivariate Cox-regression models, and restrictive cubic spline fits for obesity indexes were used in the upper panel and linear fits were used in the lower panel. Image A and B in each upper and lower panel are 90-degree rotational views of one another.
When both BMI and WC were included in the models, the risk of MACE was only associated with WC, whereas the risk of CD/NFMI was not associated with both WC and BMI. There were no significant interactions between BMI and WC in the regression models for the risk of CD/NFMI (*p* = 0.488) and MACE (*p* = 0.667).
* Adjusted for age, sex, diabetes mellitus, hypertension, current smoking, chronic kidney disease, history of PCI, STEMI, stent type (1st vs. 2nd generation), center, TSL, average stent diameter, complete revascularization and the duration of DAPT. Each model was reduced through a backward variable selection process.
CD, cardiac death; NFMI, nonfatal myocardial infarction; MACE, major adverse cardiac event; BMI body mass index; and WC, waist circumference; *df*, degrees of freedom.
(TIF)

S1 Table Baseline characteristics of the study population by the groups based on BMI and those based on WC. Data were shown in mean ± SD or n (%).
Data with a skewed distribution were presented with median (1st quartile value and 3rd quartile value)
BMI; body mass index; WC, waist circumference; PCI, percutaneous coronary intervention; LDL, low density lipoprotein; HDL, high density lipoprotein; hsCRP, higly sensitive C-reactive protein; eGFR, estimated glomerular filtration rate; LMCA, left main coronary artery; LAD; left anterior descending artery; LCX, left circumflex artery; RCA right coronary artery; DES, drug eluting stent.
(DOCX)

S2 Table Overall frequency of clinical outcomes after PCI. Data are shown as number (%).
* Median (the first quartile, the third quartile)
† Cardiac death + Non-fatal MI + TVR
PCI, percutaneous coronary intervention; BMI, body mass index; WC, waist circumference; MI, myocardial infarction; TVR, target vessel revascularization; MACE, major adverse cardiac event.
(DOCX)

S3 Table Multivariate Cox-regression analysis for both BMI and WC on the clinical outcomes. Models were reduced by a backward variable selection process (cut-off point, p <0.05) and BMI and WC were set to remain in the final model.
* Restrictive cubic spline fits for BMI and WC were used in the model (df = 4).
CD, cardiac death; NFMI, non-fatal myocardial infarction; MACE, major adverse cardiac event; BMI, body mass index; WC, waist circumference; CKD, chronic kidney disease; eGFR, estimated glomerular filtration rate; DAPT, dual antiplatelet agent therapy.
(DOCX)

S4 Table Interaction between BMI and WC on the clinical outcomes. Models were reduced by a backward variable selection process (cut-off point, p <0.05) and BMI and WC were set to remain in the final model.
* Restrictive cubic spline fits for BMI and WC were used in the model (df = 4).
CD, cardiac death; NFMI, non-fatal myocardial infarction; MACE, major adverse cardiac event; BMI, body mass index; WC, waist circumference; CKD, chronic kidney disease; eGFR, estimated glomerular filtration rate; DAPT, dual antiplatelet agent therapy.
(DOCX)
