# Supplementary figures and images for: Relationship of body mass index and waist circumference with clinical outcomes following percutaneous coronary intervention
Source: PLoS One. 2018 Dec 13;13(12):e0208817. doi: 10.1371/journal.pone.0208817 (PMC6292633; doi:10.1371/journal.pone.0208817)

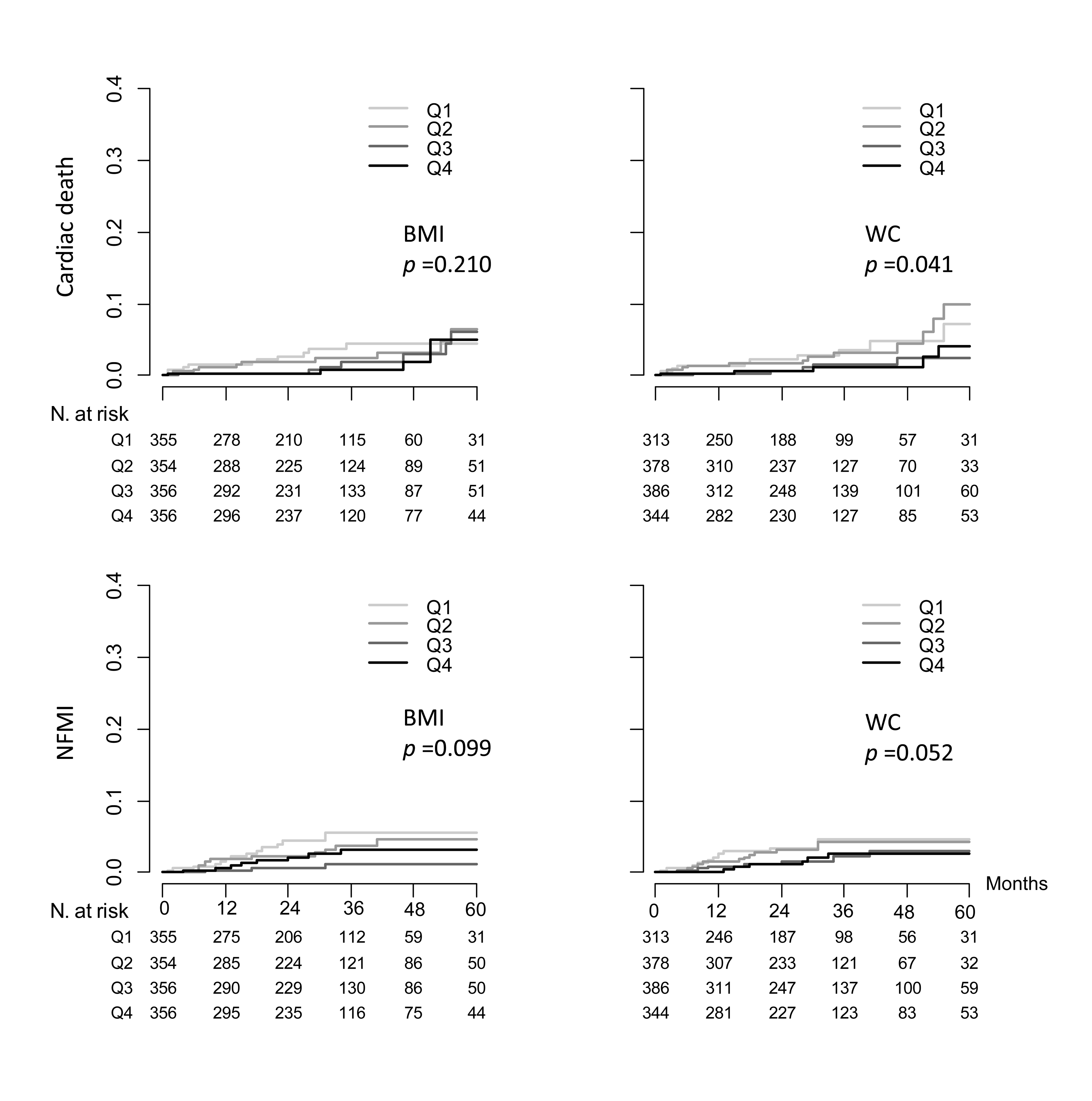

Supplement: S1 Fig — CD occurred more frequently in the high WC groups (Q3WC and Q4WC) than in the low WC groups (Q1WC and Q2WC), whereas there was no difference in the risk of CD among the groups, according to BMI. The incidence of NFMI was only marginally higher in the low WC and BMI groups. CD, cardiac death; NFMI, nonfatal myocardial infarction; BMI body mass index; WC, waist circumference. (TIF) [file pone.0208817.s001.tif]

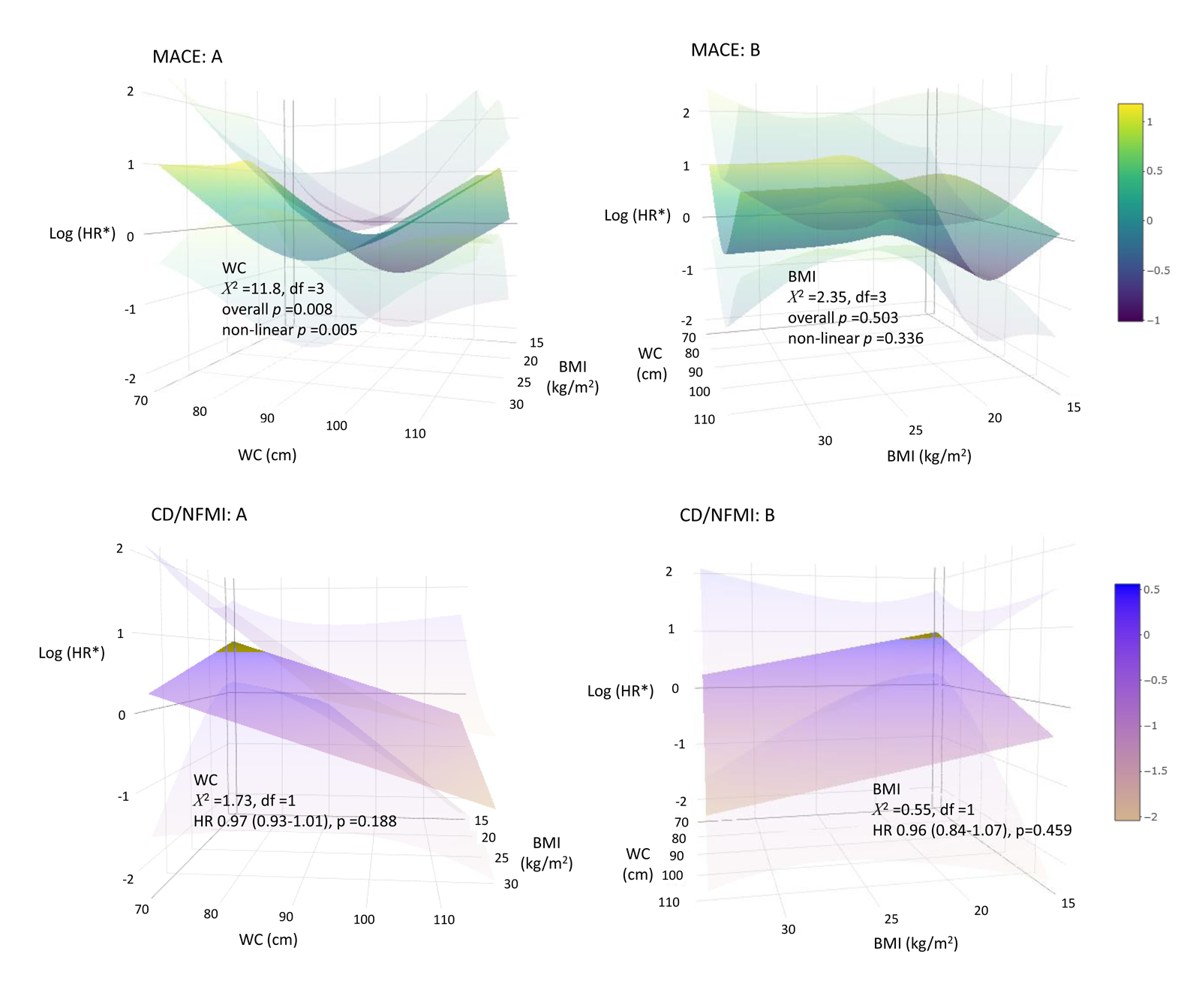

Supplement: S2 Fig — The opaquer sheaths represent the surfaces of log (HR) from multivariate Cox-regression, and the less opaque sheaths represent the surfaces of upper and lower 95% confidence interval limits. The upper panel (green sheaths) represents the risk of MACE, and the lower panel (purple) represents the risk of CD/NFMI. HRs were derived from multivariate Cox-regression models, and restrictive cubic spline fits for obesity indexes were used in the upper panel and linear fits were used in the lower panel. Image A and B in each upper and lower panel are 90-degree rotational views of one another. When both BMI and WC were included in the models, the risk of MACE was only associated with WC, whereas the risk of CD/NFMI was not associated with both WC and BMI. There were no significant interactions between BMI and WC in the regression models for the risk of CD/NFMI (p = 0.488) and MACE (p = 0.667). * Adjusted for age, sex, diabetes mellitus, hypertension, current smoking, chronic kidney disease, history of PCI, STEMI, stent type (1st vs. 2nd generation), center, TSL, average stent diameter, complete revascularization and the duration of DAPT. Each model was reduced through a backward variable selection process. CD, cardiac death; NFMI, nonfatal myocardial infarction; MACE, major adverse cardiac event; BMI body mass index; and WC, waist circumference; df, degrees of freedom. (TIF) [file pone.0208817.s002.tif]
