# Supplementary material for: Relationship of body mass index and waist circumference with clinical outcomes following percutaneous coronary intervention
Source: PLoS One. 2018 Dec 13;13(12):e0208817. doi: 10.1371/journal.pone.0208817 (PMC6292633; doi:10.1371/journal.pone.0208817)
Supplement: S1 Table — Data were shown in mean ± SD or n (%). Data with a skewed distribution were presented with median (1st quartile value and 3rd quartile value) BMI; body mass index; WC, waist circumference; PCI, percutaneous coronary intervention; LDL, low density lipoprotein; HDL, high density lipoprotein; hsCRP, higly sensitive C-reactive protein; eGFR, estimated glomerular filtration rate; LMCA, left main coronary artery; LAD; left anterior descending artery; LCX, left circumflex artery; RCA right coronary artery; DES, drug eluting stent. (DOCX) [file pone.0208817.s003.docx]

**Supporting Information**

| S1 Table. Baseline characteristics of the study population by the groups based on BMI and those based on WC | | | | | | | | | | |
| --- | --- | --- | --- | --- | --- | --- | --- | --- | --- | --- |
|  | BMI (kg/m^2^) | | | | | WC (cm) | | | | |
|  | Q1_BMI_ | Q2_BMI_ | Q3_BMI_ | Q4_BMI_ |  | Q1_WC_ | Q2_WC_ | Q3_WC_ | Q4_WC_ |  |
|  | < 22.9 | 22.9-24.8 | 24.8-26.8 | > 26.8 |  | < 84 | 84-89 | 90-94 | > 95 |  |
|  | 355 | 354 | 356 | 356 | *p*-value | 313 | 378 | 386 | 344 | *p*-value |
| Age (years) | 66 ± 11 | 63 ± 11 | 63 ± 10 | 62 ± 11 | <0.001 | 64 ± 12 | 64 ± 10 | 63 ± 10 | 64 ± 11 | 0.307 |
| Female sex | 124 (34.9) | 108 (30.5) | 111 (31.2) | 129 (36.2) | 0.288 | 120 (38.3) | 123 (32.5) | 122 (31.6) | 107 (31.1) | 0.073 |
| Diabetes mellitus | 102 (28.7) | 116 (32.8) | 116 (32.6) | 131 (36.8) | 0.154 | 79 (25.2) | 113 (29.9) | 142 (36.8) | 131 (38.1) | <0.001 |
| Hypertension | 190 (53.5) | 214 (60.5) | 226 (63.5) | 249 (69.9) | <0.001 | 157 (50.2) | 224 (59.3) | 266 (68.9) | 232 (67.4) | <0.001 |
| Chronic kidney disease | 66 (18.6) | 71 (20.1) | 43 (12.1) | 56 (15.7) | 0.023 | 58 (18.5) | 70 (18.5) | 58 (15.0) | 50 (14.5) | 0.308 |
| Current smoking | 100 (28.2) | 100 (28.2) | 88 (24.7) | 79 (22.2) | 0.188 | 83 (26.5) | 110 (29.1) | 91 (23.6) | 83 (24.1) | 0.292 |
| Past history of PCI | 47 (13.2) | 64 (18.1) | 54 (15.2) | 68 (19.1) | 0.131 | 41 (13.1) | 71 (18.8) | 64 (16.6) | 57 (16.6) | 0.254 |
| Clinical diagnosis |  |  |  |  |  |  |  |  |  |  |
| Stable angina | 86 (24.2) | 92 (26.0) | 93 (26.1) | 128 (36.0) | 0.016 | 82 (26.2) | 104 (27.4) | 114 (29.7) | 99 (28.8) | 0.852 |
| Unstable angina | 189 (53.2) | 189 (53.4) | 192 (53.9) | 174 (48.9) |  | 164 (52.4) | 202 (53.2) | 200 (52.1) | 178 (51.7) |  |
| NSTEMI | 51 (14.4) | 44 (12.4) | 52 (14.6) | 36 (10.1) |  | 39 (12.5) | 49 (12.9) | 47 (12.2) | 48 (14.0) |  |
| STEMI | 29 (8.2) | 29 (8.2) | 19 (5.3) | 18 (5.1) |  | 28 (8.9) | 25 (6.6) | 23 (6.0) | 19 (5.5) |  |
| Laboratory tests |  |  |  |  |  |  |  |  |  |  |
| Hemoglobin (g/dl) | 13.0 ± 1.8 | 13.3 ±1.8 | 13.6 ± 1.8 | 13.7 ± 1.9 | <0.001 | 12.9 ± 1.8 | 13.4 ±1.8 | 13.6 ± 1.8 | 13.6 ± 1.7 | <0.001 |
| Total cholesterol (mg/dl) | 165 ± 41 | 166 ± 39 | 168 ± 41 | 173 ± 39 | 0.004 | 169 ± 41 | 164 ± 40 | 166 ± 40 | 173 ± 40 | 0.004 |
| LDL cholesterol (mg/dl) | 94 (76, 116) | 94 (70, 119) | 97 (75, 117) | 102 (77, 120) | 0.063 | 97 (76, 121) | 94 (72, 116) | 92 (74, 115) | 102 (78, 12) | 0.003 |
| HDL cholesterol (mg/dl) | 41 (35, 49) | 41 (34, 47) | 39 (34, 46) | 40 (34, 48) | 0.153 | 42 (36, 50) | 40 (34, 47) | 40 (35, 46) | 39 (34, 48) | 0.007 |
| Triglyceride (mg/dl) | 99 (71, 140) | 117 (82, 167) | 127 (93, 180) | 128 (95, 183) | <0.001 | 98 (72, 140) | 112 (81, 166) | 121 (91, 177) | 135 (95, 185) | <0.001 |
| HgA1c (%) | 6.0 (5.5, 6.6) | 6.2 (5.7, 6.9) | 6.1 (5.7, 6.9) | 6.2 (5.8, 7.1) | <0.001 | 5.9 (5.5, 6.5) | 6.1 (5.6, 6.8) | 6.2 (5.7, 7.0) | 6.3 (5.8, 7.2) | <0.001 |
| hsCRP (mg/dl) | 0.12 (0.03, 0.46) | 0.12 (0.05, 0.37) | 0.12 (0.05, 0.45) | 0.12 (0.05, 0.35) | 0.774 | 0.11 (0.04, 0.42) | 0.11 (0.04, 0.39) | 0.12 (0.05, 0.41) | 0.15 (0.06, 0.43) | 0.179 |
| eGFR (ml/min/1.73m^2^) | 76 ± 20 | 77 ±19 | 79 ± 17 | 77 ± 17 | 0.127 | 77 ± 20 | 77 ±18 | 78 ± 18 | 77 ± 17 | 0.342 |
| LVMI (g/m^2^) | 103 ± 30 | 107 ± 10 | 109 ± 31 | 111 ± 32 | <0.001 | 103 ± 29 | 106 ± 30 | 108 ± 30 | 112 ± 32 | <0.001 |
| Ejection Fraction (%) | 66.2 ± 11.3 | 66.5 ± 10.4 | 67.7 ± 10.4 | 68.5 ± 9.8 | 0.001 | 66.5 ± 11.2 | 67.0 ± 9.9 | 67.7 ± 10.8 | 67.8 ± 10.1 | 0.078 |
| Post PCI medications |  |  |  |  |  |  |  |  |  |  |
| Statin | 292 (82.3) | 300 (84.7) | 291 (81.7) | 313 (87.9) | 0.094 | 258 (82.4) | 313 (82.8) | 322 (83.4) | 303 (88.1) | 0.148 |
| Angiotensin blockers | 250 (70.4) | 262 (74.0) | 264 (74.2) | 276 (77.5) | 0.197 | 227 (72.9) | 255 (67.5) | 302 (78.2) | 268 (77.9) | 0.002 |
| Beta-blockers | 221 (62.3) | 211 (59.6) | 216 (60.7) | 246 (69.1) | 0.040 | 190 (60.7) | 237 (62.7) | 242 (62.7) | 225 (65.4) | 0.662 |
| Duration of DAPT (months) | 18.0 (9.0, 33.0) | 17.0 (10.0, 32.0) | 20.0 (11.0, 34.0) | 19.0 (12.0, 34.0) | 0.083 | 16.0 (9.0, 31.0) | 21.0 (10.0, 34.0) | 19.0 (11.8, 34.0) | 19.0 (12.0, 33.0) | 0.047 |
| Number of coronary arteries narrowed | |  |  |  |  |  |  |  |  |  |
| 1 | 202 (56.9) | 207 (58.5) | 196 (55.1) | 217 (61.0) | 0.343 | 182 (58.1) | 215 (56.9) | 233 (60.4) | 192 (55.8) | 0.586 |
| 2 | 116 (32.7) | 101 (28.5) | 108 (30.3) | 104 (29.2) |  | 98 (31.3) | 115 (30.4) | 114 (29.5) | 102 (29.7) |  |
| 3 | 37 (10.4) | 46 (13.0) | 52 (14.6) | 35 (9.8) |  | 33 (10.5) | 48 (12.7) | 39 (10.1) | 50 (14.5) |  |
| Coronary arteries involved |  |  |  |  |  |  |  |  |  |  |
| LMCA | 31 (8.7) | 17 (4.8) | 14 (3.9) | 22 (6.7) | 0.038 | 19 (6.1) | 28 (7.4) | 18 (4.7) | 19 (5.5) | 0.439 |
| LAD | 251 (70.7) | 254 (71.8) | 261 (73.3) | 230 (64.6) | 0.060 | 215 (68.7) | 274 (72.5) | 269 (69.7) | 238 (69.2) | 0.684 |
| LCX | 110 (31.0) | 131 (37.0) | 138 (38.8) | 126 (35.4) | 0.160 | 99 (31.6) | 132 (34.9) | 135 (35.0) | 139 (40.4) | 0.124 |
| RCA | 152 (42.8) | 144 (40.7) | 154 (43.3) | 146 (41.0) | 0.868 | 140 (44.7) | 153 (40.5) | 154 (39.9) | 149 (43.3) | 0.520 |
| Complete revascularization | 278 (88.8) | 343 (90.3) | 339 (88.3) | 298 (86.9) | 0.553 | 318 (89.6) | 309 (87.3) | 311 (87.4) | 320 (90.1) | 0.510 |
| Number of stents implanted |  |  |  |  |  |  |  |  |  |  |
| 1 | 157 (44.2) | 166 (46.9) | 147 (41.3) | 179 (50.3) | 0.188 | 150 (47.9) | 168 (44.4) | 182 (47.2) | 149 (43.3) | 0.362 |
| 2 | 104 (29.3) | 89 (25.1) | 97 (27.2) | 80 (22.5) |  | 90 (28.8) | 95 (25.1) | 97 (25.1) | 88 (25.6) |  |
| ≥3 | 94 (26.5) | 99 (28.0) | 112 (31.5) | 97 (27.2) |  | 73 (23.3) | 115 (30.5) | 107 (27.7) | 107 (31.1) |  |
| Average stent diameters (mm) | 3.2 ± 0.4 | 3.2 ± 0.4 | 3.2 ± 0.4 | 3.2 ± 0.4 | 0.573 | 3.2 ± 0.4 | 3.2 ± 0.4 | 3.2 ± 0.4 | 3.2 ± 0.4 | 0.911 |
| Total stent length (mm) | 43 (24, 71) | 40 (24, 69) | 46 (24, 76) | 38 (24, 69) | 0.417 | 38 (24, 64) | 44 (24, 76) | 38 (23, 71) | 44 (28, 75) | 0.281 |
| Types of stents |  |  |  |  |  |  |  |  |  |  |
| Paclitaxel | 49 (13.8) | 59 (16.7) | 54 (15.2) | 49 (13.8) | 0.401 | 48 (15.3) | 60 (15.9) | 54 (14.0) | 49 (14.2) | 0.964 |
| Sirolimus | 72 (20.3) | 91 (25.7) | 80 (22.5) | 71 (19.9) |  | 74 (23.6) | 77 (20.4) | 91 (23.6) | 72 (20.9) |  |
| Everolimus | 231 (65.1) | 202 (51.7) | 218 (61.2) | 230 (64.6) |  | 188 (60.1) | 236 (62.4) | 238 (61.7) | 219 (63.7) |  |
| Second generation DES | 233 (65.6) | 204 (57.6) | 224 (62.9) | 234 (64.7) | 0.087 | 189 (60.4) | 239 (63.2) | 243 (63.0) | 224 (65.1) | 0.662 |
| Data were shown in mean ± SD or n (%).  Data with a skewed distribution were presented with median (1st quartile value and 3rd quartile value)  BMI; body mass index; WC, waist circumference; PCI, percutaneous coronary intervention; LDL, low density lipoprotein; HDL, high density lipoprotein; hsCRP, higly sensitive C-reactive protein; eGFR, estimated glomerular filtration rate; LMCA, left main coronary artery; LAD; left anterior descending artery; LCX, left circumflex artery; RCA right coronary artery; DES, drug eluting stent. | | | | | | | | | | |
