# Supplementary material for: Relationship of body mass index and waist circumference with clinical outcomes following percutaneous coronary intervention
Source: PLoS One. 2018 Dec 13;13(12):e0208817. doi: 10.1371/journal.pone.0208817 (PMC6292633; doi:10.1371/journal.pone.0208817)
Supplement: S2 Table — Data are shown as number (%). * Median (the first quartile, the third quartile) † Cardiac death + Non-fatal MI + TVR PCI, percutaneous coronary intervention; BMI, body mass index; WC, waist circumference; MI, myocardial infarction; TVR, target vessel revascularization; MACE, major adverse cardiac event. (DOCX) [file pone.0208817.s004.docx]

**Supporting Information**

| S2 Table. Overall frequency of clinical outcomes after PCI | | | | | | | | | | |
| --- | --- | --- | --- | --- | --- | --- | --- | --- | --- | --- |
|  | BMI | | | | | WC | | | | |
|  | Q1_BMI_ | Q2_BMI_ | Q3_BMI_ | Q4_BMI_ |  | Q1_WC_ | Q2_WC_ | Q3_WC_ | Q4_WC_ |  |
|  | 355 | 354 | 356 | 356 | *p*-value | 313 | 378 | 386 | 344 | *p*-value |
| Follow-up duration (months) | 28 (11, 37) | 29 (12, 43) | 31 (13, 44) | 29 (15, 40) | 0.221 | 26 (11, 36) | 31 (13, 39) | 31 (15, 46) | 29 (13, 43) | 0.097 |
| Cardiac death | 11 (3.1) | 10 (2.8) | 7 (2.0) | 5 (1.4) | 0.414 | 11 (3.5) | 13 (3.4) | 5 (1.3) | 5 (1.5) | 0.084 |
| Non-fatal MI | 14 (3.9) | 11 (3.1) | 5 (1.4) | 7 (2.0) | 0.139 | 12 (3.8) | 14 (3.7) | 5 (1.3) | 6 (1.7) | 0.066 |
| TVR | 40 (11.3) | 48 (13.6) | 46 (12.9) | 50 (14.0) | 0.709 | 52 (16.6) | 44 (11.6) | 35 (9.1) | 54 (15.7) | 0.009 |
| MACE † | 64 (18.0) | 64 (18.1) | 52 (14.6) | 61 (17.1) | 0.570 | 71 (22.7) | 66 (17.4) | 42 (10.9) | 64 (18.6) | <0.001 |
| Data are shown as number (%).  * Median (the first quartile, the third quartile)  † Cardiac death + Non-fatal MI + TVR  PCI, percutaneous coronary intervention; BMI, body mass index; WC, waist circumference; MI, myocardial infarction; TVR, target vessel revascularization; MACE, major adverse cardiac event. | | | | | | | | | | |
