# Supplementary material for: Relationship of body mass index and waist circumference with clinical outcomes following percutaneous coronary intervention
Source: PLoS One. 2018 Dec 13;13(12):e0208817. doi: 10.1371/journal.pone.0208817 (PMC6292633; doi:10.1371/journal.pone.0208817)
Supplement: S3 Table — Models were reduced by a backward variable selection process (cut-off point, p <0.05) and BMI and WC were set to remain in the final model. * Restrictive cubic spline fits for BMI and WC were used in the model (df = 4). CD, cardiac death; NFMI, non-fatal myocardial infarction; MACE, major adverse cardiac event; BMI, body mass index; WC, waist circumference; CKD, chronic kidney disease; eGFR, estimated glomerular filtration rate; DAPT, dual antiplatelet agent therapy. (DOCX) [file pone.0208817.s005.docx]

**Supporting Information**

| S3 Table. Multivariate Cox-regression analysis for both BMI and WC on the clinical outcomes. | | | | | | | | |
| --- | --- | --- | --- | --- | --- | --- | --- | --- |
| Model summary | Variables | coefficient | S.E | HR | 95% CI | χ^2^ | d.f. | *p* |
| CD/NFMI | BMI | -0.040 | 0.054 | 0.96 | 0.86-1.07 | 0.55 | 1 | 0.4588 |
| C-statistic 0.853 | WC | -0.029 | 0.022 | 0.97 | 0.93-1.01 | 1.73 | 1 | 0.1882 |
|  | Diabetes mellitus | 0.713 | 0.255 | 2.04 | 1.24-3.36 | 7.82 | 1 | 0.0052 |
|  | Complete revascularization | -0.908 | 0.295 | 0.40 | 0.23-0.72 | 9.46 | 1 | 0.0021 |
|  | eGFR (mL/min/1.72 m^2^) | -0.028 | 0.006 | 0.97 | 0.96-0.98 | 21.76 | 1 | <0.0001 |
|  | Duration of DAPT (months) | -0.079 | 0.012 | 0.92 | 0.90-0.95 | 42.82 | 1 | <0.0001 |
| MACE* | BMI overall | - | - | - | - | 2.51 | 3 | 0.4733 |
| C-statistic 0.845 | non-linear | - | - | - | - | 2.23 | 2 | 0.3278 |
|  | WC overall | - | - | - | - | 11.73 | 3 | 0.0084 |
|  | non-linear | - | - | - | - | 10.02 | 2 | 0.0067 |
|  | Male sex | 0.390 | 0.150 | 1.48 | 1.10-1.98 | 6.79 | 1 | 0.0092 |
|  | Diabetes mellitus | 0.438 | 0.137 | 1.55 | 1.18-2.03 | 10.29 | 1 | 0.0013 |
|  | Total stent length (per 10 mm) | 0.064 | 0.016 | 1.07 | 1.03-1.10 | 15.06 | 1 | 0.0001 |
|  | Center 1 | -1.062 | 0.210 | 0.35 | 0.23-0.52 | 25.54 | 1 | <0.0001 |
|  | Second generation DES | -1.211 | 0.197 | 0.30 | 0.20-0.44 | 37.87 | 1 | <0.0001 |
|  | Duration of DAPT (months) | -0.072 | 0.006 | 0.93 | 0.92-0.94 | 143.28 | 1 | <0.0001 |
| Models were reduced by a backward variable selection process (cut-off point, p <0.05) and BMI and WC were set to remain in the final model. | | | | | | | | |
| * Restrictive cubic spline fits for BMI and WC were used in the model (df=4). | | | | | | | | |
| CD, cardiac death; NFMI, non-fatal myocardial infarction; MACE, major adverse cardiac event; BMI, body mass index; WC, waist circumference; CKD, chronic kidney disease; eGFR, estimated glomerular filtration rate ; DAPT, dual antiplatelet agent therapy. | | | | | | | | |
