# Supplementary material for: Relationship of body mass index and waist circumference with clinical outcomes following percutaneous coronary intervention
Source: PLoS One. 2018 Dec 13;13(12):e0208817. doi: 10.1371/journal.pone.0208817 (PMC6292633; doi:10.1371/journal.pone.0208817)
Supplement: S4 Table — Models were reduced by a backward variable selection process (cut-off point, p <0.05) and BMI and WC were set to remain in the final model. * Restrictive cubic spline fits for BMI and WC were used in the model (df = 4). CD, cardiac death; NFMI, non-fatal myocardial infarction; MACE, major adverse cardiac event; BMI, body mass index; WC, waist circumference; CKD, chronic kidney disease; eGFR, estimated glomerular filtration rate; DAPT, dual antiplatelet agent therapy. (DOCX) [file pone.0208817.s006.docx]

**Supporting Information**

| S4 Table. Interaction between BMI and WC on the clinical outcomes. | | | | | | | | |
| --- | --- | --- | --- | --- | --- | --- | --- | --- |
| Model summary | Variables | coefficient | S.E | HR | 95% CI | χ^2^ | d.f. | *p* |
| CD/NFMI | BMI | 0.259 | 0.435 | 1.30 | 0.55-3.04 | 1.04 | 2 | 0.5944 |
| C-statistic 0.855 | WC | 0.053 | 0.120 | 1.05 | 0.83-1.33 | 2.20 | 2 | 0.3326 |
|  | BMI*WC | -0.003 | 0.005 | 1.00 | 0.99-1.01 | 0.48 | 1 | 0.4879 |
|  | Diabetes mellitus | 0.715 | 0.255 | 2.04 | 1.24-3.37 | 7.82 | 1 | 0.0052 |
|  | Complete revascularization | -0.921 | 0.296 | 0.40 | 0.22-0.71 | 9.46 | 1 | 0.0021 |
|  | eGFR (mL/min/1.72 m^2^) | -0.028 | 0.006 | 0.97 | 0.96-0.98 | 21.76 | 1 | <0.0001 |
|  | Duration of DAPT (months) | -0.079 | 0.012 | 0.92 | 0.90-0.95 | 42.82 | 1 | <0.0001 |
| MACE* | BMI overall | - | - | - | - | 9.00 | 12 | 0.7034 |
| C-statistic 0.845 | non-linear | - | - | - | - | 7.59 | 8 | 0.4749 |
|  | WC overall | - | - | - | - | 18.29 | 12 | 0.1071 |
|  | non-linear | - | - | - | - | 10.11 | 8 | 0.2574 |
|  | BMI*WC | - | - | - | - | 6.71 | 9 | 0.6672 |
|  | Male sex | 0.389 | 0.150 | 1.47 | 1.10-1.98 | 6.71 | 1 | 0.0092 |
|  | Diabetes mellitus | 0.458 | 0.137 | 1.58 | 1.21-2.07 | 11.19 | 1 | 0.0013 |
|  | Total stent length (per 10 mm) | 0.066 | 0.017 | 1.07 | 1.03-1.10 | 15.77 | 1 | 0.0001 |
|  | Center 1 | -1.07 | 0.211 | 0.34 | 0.23-0.52 | 25.60 | 1 | <0.0001 |
|  | Second generation DES | -1.218 | 0.198 | 0.30 | 0.20-0.44 | 38.01 | 1 | <0.0001 |
|  | Duration of DAPT (months) | -0.074 | 0.006 | 0.93 | 0.92-0.94 | 144.6 | 1 | <0.0001 |
| Models were reduced by a backward variable selection process (cut-off point, p <0.05) and BMI and WC were set to remain in the final model. | | | | | | | | |
| * Restrictive cubic spline fits for BMI and WC were used in the model (df=4). | | | | | | | | |
| CD, cardiac death; NFMI, non-fatal myocardial infarction; MACE, major adverse cardiac event; BMI, body mass index; WC, waist circumference; CKD, chronic kidney disease; eGFR, estimated glomerular filtration rate; DAPT, dual antiplatelet agent therapy. | | | | | | | | |
